# Transplant Trial Watch

**DOI:** 10.3389/ti.2025.14375

**Published:** 2025-02-18

**Authors:** Simon R. Knight, John Fallon

**Affiliations:** ^1^ Nuffield Department of Surgical Sciences, Centre for Evidence in Transplantation, University of Oxford, Oxford, United Kingdom; ^2^ Oxford Transplant Centre, Churchill Hospital, Oxford, United Kingdom

**Keywords:** randomised controlled trial, systematic review/Meta-analysis, liver transplantation (LT), kidney transplantation (KT), BK polyomavirus

## Aims

To comprehensively evaluate whether hypothermic oxygenated perfusion (HOPE) offers significant benefits over static cold storage (SCS) in adult liver transplantation, focusing on graft outcomes, complications, and patient prognosis.

## Interventions

The control group of donor livers preserved using SCS compared with the intervention group of donor livers preserved using HOPE.

## Participants

They included 11 studies (4 RCTs, 4 prospective non-randomized, 3 retrospective), totalling 1,765 adult liver transplant recipients: HOPE in 532 patients and SCS in 1,233 patients. Donor grafts included donation after brain death (DBD), extended criteria donor DBD, and donation after circulatory death (DCD).

## Outcomes

Primary Outcomes: early allograft dysfunction (EAD), primary non-function (PNF), acute rejection and one-year graft loss Secondary Outcomes: one-year mortality, biliary complications, vascular complications, major postoperative complications (Clavien-Dindo grade ≥ IIIa or ≥ IIIb) and additional descriptive outcomes (peak liver enzymes, ICU/hospital stay) where reported.

## Follow-Up

Follow-up varied across studies, with most tracking outcomes up to one-year post-transplant.

## CET Conclusion

*by John Fallon*

The authors conducted a robust and comprehensive systematic review and meta-analysis, with 11 studies of at least moderate quality, 4 small to moderate sized RCTs, 3 of which were multi-centre and 7 cohort studies on of which was a large retrospective study with 121 livers having undergone HOPE. The analyses demonstrate a significant reduction in EAD: HOPE substantially decreased early allograft dysfunction (pooled OR ∼0.36) and A lower graft loss rate: one-year graft loss was significantly less frequent with HOPE (pooled OR ∼0.57). With regards complication profiles HOPE was associated with fewer Clavien-Dindo ≥IIIa complications and tended to reduce biliary complications, acute rejection, and vascular complications (though sensitivity analyses revealed some heterogeneity among studies). As has been seen in the kidney and is consistent among liver studies HOPE is particularly beneficial for DCD Grafts: Subgroup analysis showed HOPE recipients with DCD grafts had reduced biliary complications, one-year mortality, and acute rejection. As with all analyses of this nature, they are limited by the quality of underlying studies, in this case the are a reasonable number of randomised studies, and across the studies a low heterogeneity for certain important outcomes such EAD which provides strong evidence. There is, of course, moderate of high levels for some complications due to variability in study design and populations, however this does not weaken the overall message. Overall, the evidence supports a notable advantage of HOPE in reducing ischemia-reperfusion injury and improving early and some longer-term outcomes in liver transplantation, especially for higher-risk grafts such as DCD. This being said, there is no large multi-centre/multi-national RCT which could definitively demonstrate the need for ubiquitous HOPE, especially in marginal grafts.

## Trial Registration

PROSPERO - CRD4202343074.

## Funding Source

Non-industry funded.

## Aims

This study aimed to examine whether the administration of everolimus (EVR) was more effective in facilitating the clearance of BK polyomavirus (BKPyV) infection in comparison to standard immunosuppression reduction in kidney transplant recipients.

## Interventions

Participants were randomised to either the mycophenolate mofetil (MMF) group or the EVR group.

## Participants

130 kidney transplant recipients.

## Outcomes

The primary outcome was the proportion of patients that were able to achieve BKPyV clearance. The secondary outcomes were the assessment of BKPyV replication kinetics, the incidence of biopsy-proven BKPyVN, rate of rejection, change in kidney allograft function, the incidence of donor-specific antibodies (DSAs) and treatment safety.

## Follow-Up

2 years following randomisation.

## CET Conclusion

*by Simon Knight*

This multicentre randomised controlled trial investigated the role of everolimus in the management of kidney transplant recipients with BK virus infection. 130 kidney recipients with BK viraemia were randomised to standard immunosuppression reduction versus a switch from MMF to everolimus. BK virus clearance was actually higher in the MMF arm, despite similar CNI trough levels. This is an interesting and well-designed study, although a lack of blinding and a fixed randomisation block size might have affected allocation concealment. Intent-to-treat analysis is used. It should be noted that patients with established BK virus nephropathy were excluded. Given the antiviral properties of mTOR inhibitors, the results are surprising. The authors hypothesise that higher overall immunosuppression or insufficient levels of everolimus to exert an antiviral effect may provide an explanation.

## Jadad Score

3.

## Data Analysis

Strict intention-to-treat analysis.

## Allocation Concealment

Yes.

## Trial Registration

ClinicalTrials.gov - NCT03216967.

## Funding Source

Non-industry funded.

## Clinical Impact Summary

*by Simon Knight*

Previous studies have suggested that mammalian target of rapamycin inhibitors (mTORi) may have antiviral properties, potentially giving them a role in management of infections post-transplant [1]. mTORi enhance the quantity and quality of memory CD8 T-cells following viral infection or vaccination [2], and when used *de novo* in kidney transplant recipients appear to reduce the risk of CMV and BK viral infection [3].

The role of mTORi in the management of established viral infection post-transplant is less clear. Current management of BK virus post-transplant centres around reduction in immunosuppression, with no compelling evidence for the use of antiviral agents [4].

The multicentre BKEVER trial investigated the efficacy of switching from mycophenolate mofetil (MMF) to everolimus, with reduced dose calcineurin inhibitor (CNI), compared to standard MMF and CNI reduction in kidney transplant recipients with BK viraemia [5]. 130 patients were randomised across 16 transplant centres. Contrary to the author’s hypothesis, BK viral clearance was actually higher in the MMF group at 6 months (81.3% vs. 55.7%) with numerically higher rejection rates in the everolimus group and no difference in graft survival.

These results are difficult to explain, but the authors postulate that there may have still been a higher overall immunosuppressive load in the everolimus group despite similar trough CNI levels. The frequency of rejection episodes would argue against this. An alternative explanation is that the everolimus levels achieved were not sufficient to exert an antiviral effect.

Whatever the explanation, the results of this study suggest that the use of mTORi at the doses used in this study for the management of established BK virus infection is ineffective.

### Clinical Impact

4/5.
